# The Feasibility and Efficacy of Remote App-Guided Home Exercises for Frozen Shoulder: A Pilot Study

**DOI:** 10.3390/healthcare12111095

**Published:** 2024-05-27

**Authors:** Yi-Jun Lin, Chia-Ying Chung, Carl P. C. Chen, Yu-Wei Hsieh, Ching-Fu Wang, Chih-Chi Chen

**Affiliations:** 1 Department of Physical Medicine and Rehabilitation, Chang Gung Memorial Hospital, Chang Gung University, Taoyuan 333423, Taiwan; ivy9941016@cgmh.org.tw (Y.-J.L.); chiaying928@yahoo.com (C.-Y.C.); carlchendr@gmail.com (C.P.C.C.); 2 Department of Occupational Therapy and Graduate Institute of Behavioral Sciences, College of Medicine, Chang Gung University, Taoyuan 33302, Taiwan; ywhsieh@mail.cgu.edu.tw; 3 Department of Biomedical Engineering, National Yang Ming Chiao Tung University, Taipei 112304, Taiwan; chingfu.wang@nycu.edu.tw; 4 Biomedical Engineering Research and Development Center, National Yang Ming Chiao Tung University, Taipei 112304, Taiwan

**Keywords:** frozen shoulder, mobile application, home exercise, feasibility study, outcome

## Abstract

Home exercise programs are beneficial in managing frozen shoulder (FS), yet adherence remains challenging. This pilot study introduces the remote app, Defrozen, designed for home exercises and assesses its feasibility and clinical outcomes in FS patients undergoing intra-articular and sub-acromial corticosteroid treatment. Over a four-week period, patients used the Defrozen-app, engaging in guided exercises. The feasibility of the intervention was assessed through several measurement scales, including adherence, the Technology Acceptance Model 2 (TAM2), the System Usability Scale (SUS), and User Satisfaction and Engagement (USE). Clinical outcomes included pain scale, Oxford Shoulder Score (OSS), Quick Disability of the Arm, Shoulder, and Hand (QuickDASH) Score, and passive range of motion. The TAM2 results indicated high perceived usefulness (4.5/5), ease of use (4.8/5), and intention to use (4.4/5); the SUS score was high at 81.7/100, complemented by USE scores reflecting ease of learning (4.9/5) and satisfaction (4.3/5). Clinical outcomes showed significant pain reduction, improved shoulder function, reduced shoulder-related disability, and increased shoulder range of motion. These findings suggest the Defrozen-app as a promising solution for FS, significantly improving adherence and showing potential to enhance clinical outcomes. However, these clinical outcome results are preliminary and necessitate further validation through a large-scale randomized controlled trial to definitively confirm efficacy and assess long-term benefits.

## 1. Introduction

Frozen shoulder (FS) is characterized by the development of a thickened, fibrosed joint capsule, joint contraction, and reduced intra-articular volume [1]. Clinical diagnosis encompasses a variety of presentations, typically progressing through three distinct overlapping phases: the freezing phase, marked by pain and stiffness; the frozen phase, during which pain improves but shoulder range of motion progressively decreases; and the thawing phase, where range of motion gradually restores. Each phase spans several months. Despite often being considered a self-limiting condition with a natural course of 1–3 years, 40–50% of patients experience lingering clinical symptoms [2].

The lifetime prevalence of FS is estimated to range between 2% and 5% [3]. The annual cost for a patient diagnosed with frozen shoulder is estimated to be between USD 7000 and USD 8000 [4]. Additionally, the pain and limited range of motion in the affected shoulder may impair patients’ functionality, potentially reducing their productivity and leading to a heightened economic burden on both patients and their families.

A well-defined, evidence-based management guideline for FS is still lacking. The current treatment primarily centers on pain relief and the restoration of range of motion and function [5]. Shoulder exercises have demonstrated effectiveness in treating frozen shoulders. A recent meta-analysis indicates that both standalone exercise programs and those incorporating exercises contribute to improvements in range of motion, pain, and function for patients with frozen shoulders [6]. The inclusion of exercise in a treatment regimen results in greater enhancements in range of motion compared to a program without exercise. The specific types of exercises, including range of motion exercises, wall exercises, scapular exercises, and pendulum exercises, show little or no discernible difference in outcomes [6].

While one study indicated that exercise classes yielded a greater increase in range of motion and function compared to home exercises in patients with FS [7], a randomized controlled trial indicated that there was no significant difference in clinical outcomes between supervised physiotherapy in addition to home exercises and a self-directed home exercise program in patients with primary FS after hydrodilation [8]. A systematic review and meta-analysis on the management of frozen shoulder assessed the effectiveness of available strategies across 65 studies with 4097 patients. The review suggested that the early use of intra-articular corticosteroid in patients with frozen shoulders of less than 1 year duration is associated with a better outcome [9]. In our study, all the participants underwent both glenohumeral joint and subacromial space injections, guided by previous research indicating a synergistic effect of combination injections in enhancing joint internal rotation angle [10]. When accompanied by home exercise programs, these outcomes are even better than those achieved with solely intra-articular corticosteroid injections, as indicated by the results of their subgroup analysis [9]. Despite recommendations for patients with FS to regularly engage in shoulder home exercises [11], maintaining consistent adherence to these exercises proves challenging, often due to low compliance [12,13].

The strategies to enhance adherence to home exercises for patients with FS are crucial and in high demand [14]. Mobile apps have significantly transformed modern lifestyles in the past decade, with numerous applications extending into the healthcare sector [15]. The World Health Organization (WHO) categorizes these tools as electronic health (eHealth) and mobile health (m-Health) applications [16]. Apps have demonstrated their benefits in chronic pain management, included conditions such as low back pain and arthritis [17]. Although numerous studies have explored m-Health applications for the rehabilitation of the musculoskeletal system of the shoulder [10,18], the focus has predominantly been on assessing either the feasibility or usability of these apps [19,20,21], with some studies exclusively investigating clinical effects [22]. Notably, Thomas Stütz et al. evaluated the feasibility, usability, effects, and adherence in their study, demonstrating promising results; however, their small sample size of only five subjects limited the generalizability of the findings [23]. Furthermore, while Choi et al. conducted a comprehensive evaluation of the feasibility, usability, and clinical effects of an app designed for frozen shoulder rehabilitation, their study did not consider adherence rates [24], a critical factor for the success of any rehabilitation program. Our study seeks to fill these gaps by not only assessing feasibility and usability but also by rigorously monitoring and reporting adherence rates, thus providing a more complete evaluation of the intervention’s practical impact in a real-world setting [25].

In this study, we developed an easy-to-use m-Health app named Defrozen-app, which offers seven shoulder exercises covering all-direction shoulder movements to address the capsular pattern restriction of frozen shoulder. These exercises include flexion, extension, abduction, adduction, internal rotation, external rotation, and pectoral muscle stretches, chosen based on findings from a recent meta-analysis which suggests that incorporating various types of exercises, particularly static stretches, into a multimodal program may enhance range of motion [6]. The primary purpose of our app is not only to facilitate correct exercise postures and assist patients in recalling learned exercises but also to enable them to track their progress through the app’s calendar for self-reward and reminders.

The primary aim of this study is to assess the feasibility of the developed Defrozen-app intervention for patients with FS, who had undergone intra-articular and subacromial corticosteroid treatment. Feasibility was evaluated through app usability and compliance in usage. Additionally, we assessed the effects of the intervention by evaluating changes in shoulder function, pain improvement, and range of motion.

## 2. Materials and Methods

The prospective study was designed to assess the feasibility of our developed mobile health application, Defrozen-app, intended to aid patients with frozen shoulders in conducting shoulder exercises at home. This app offers seven shoulder exercises encompassing all-direction movements, including flexion, extension, abduction, adduction, internal rotation, external rotation, and pectoral muscle stretching [6]. It features a training mode with exercise demonstrations, an exercise record, and pain evaluation. We enrolled patients diagnosed with FS, who had undergone intra-articular and subacromial corticosteroid treatment for FS. During the subsequent home exercise period, participants received workout instructions through the Defrozen-app.

### 2.1. Participants

The inclusion criteria for participants encompassed unilateral frozen shoulder, suitability for intra-articular glucocorticosteroid injection, and meeting diagnostic criteria for frozen shoulder, which included shoulder pain lasting at least one month, sleep disturbance such as night pain or difficulty lying on the affected side, and limitations in both active and passive shoulder movements. The reduced passive range of motion needed to exhibit a capsular pattern, with a reduction of more than 30% in two out of three shoulder movements (abduction, internal rotation, and external rotation), and none of the three movements should be normal [17]. Plain radiographs were obtained for all participants to rule out other pathologies. The calculation of the case number was guided by the study of Julious et al., which recommended a rule of thumb of 12 cases per group for feasibility studies [18]. As we intended to compare pre-app and post-app application outcomes, our aim was to recruit a participant pool exceeding this minimum threshold of 12.

The exclusion criteria involved individuals younger than 20 years old, bilateral shoulder involvement, contraindications for anesthesia or corticosteroid infection, rheumatoid arthritis, substantial trauma history, shoulder joint injections or upper limb surgery within the past months, central nervous system disorders, cognitive disorders, incapability to learn exercise instructions, or inability to use smartphones. Patients who received treatments for the affected shoulder outside the study after enrollment were also excluded.

### 2.2. Study Flow

The research spanned a period of four weeks. Prior to participation, patients provided informed consent by signing a comprehensive patient information document, detailing the study’s objectives, the voluntary nature of participation, and the data collected through the application. Baseline characteristics and relevant outcome measures were obtained from patients before interventions. Following four weeks of interventions, feasibility and relevant outcome measures were assessed.

All the participants underwent both glenohumeral joint and subacromial space injections, guided by previous research that indicated a synergistic effect of combination injections in enhancing joint internal rotation angle [10]. The injections comprised a solution of 1 mL of 20 mg/mL triamcinolone acetonide and 1 mL of 1% lidocaine for each site [22]. 

The participants were provided with the Defrozen-app on their personal smartphones and instructed to use it daily to log their training activities. All training and assessments at home were conducted independently, without direct supervision from the physiatrist or physiotherapist.

### 2.3. Defrozen-app for Demonstrating Home Exercises for Frozen Shoulder

The Defrozen-app is designed to provide simple home exercise programs for patients with frozen shoulders, featuring a training mode with exercise demonstrations, an exercise calendar, and a pain evaluation component.

**Figure 1:**
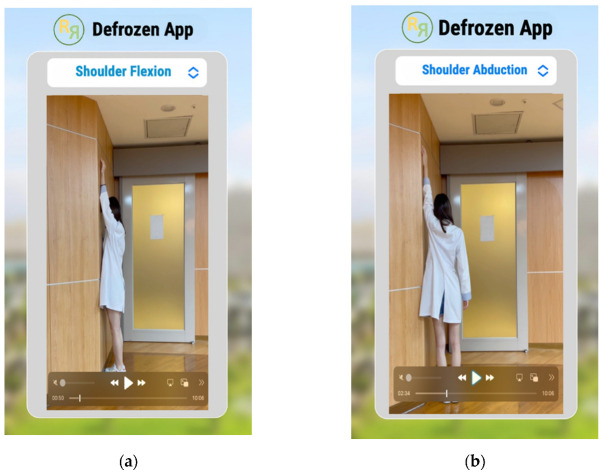

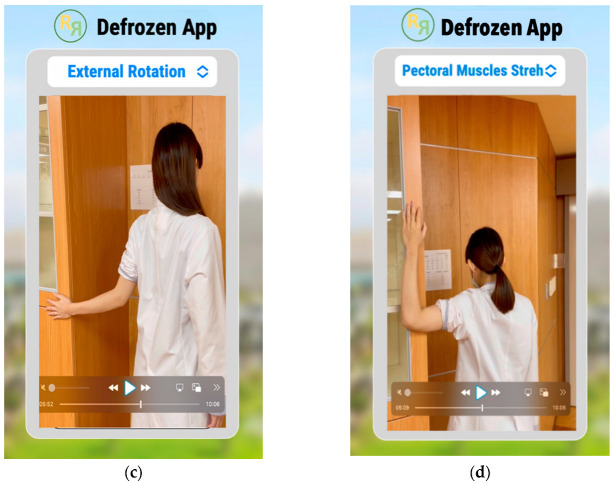

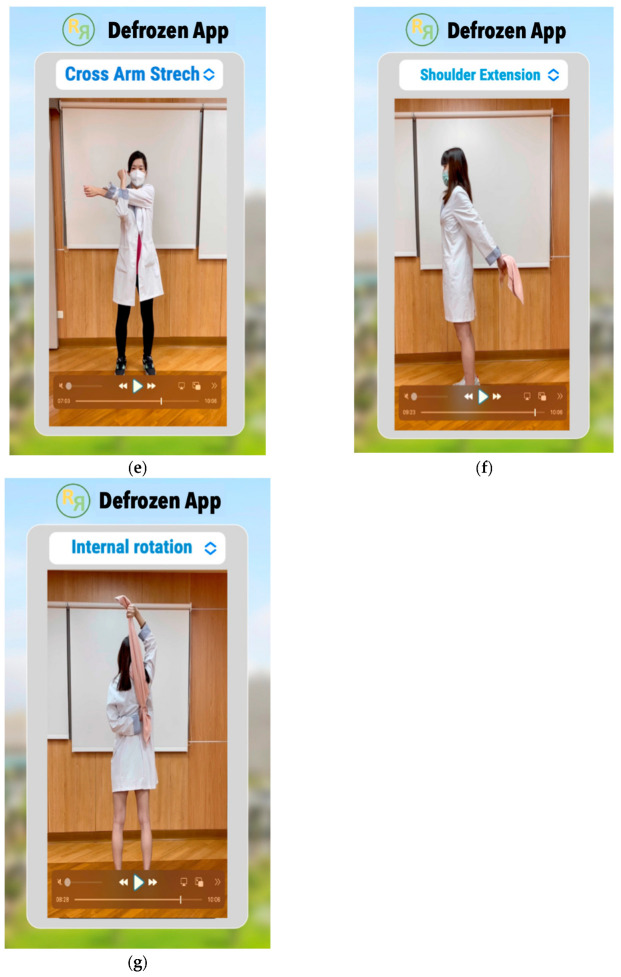
Seven sets of exercises for frozen shoulder: (**a**) finger walk for shoulder flexion; (**b**) finger walk for shoulder abduction; (**c**) self-stretching exercise for external rotation; (**d**) self-stretching exercise for pectoral muscle; (**e**) cross arm stretch for shoulder adduction; (**f**) towel exercise for shoulder extension; (**g**) towel exercise for shoulder internal rotation.

The main screen of the app showcases exercise videos led by one of our researchers, covering seven sets of exercises carefully selected by physicians and a physiotherapist from our team. These exercises include the finger walk for shoulder flexion and abduction (Figure 1a,b), self-stretching exercises for shoulder external rotation and pectoral muscles, and the crossover arm stretch for shoulder adduction (Figure 1c–e). Additionally, towel exercises for shoulder extension and internal rotation are included (Figure 1f,g).

**Figure 2:**
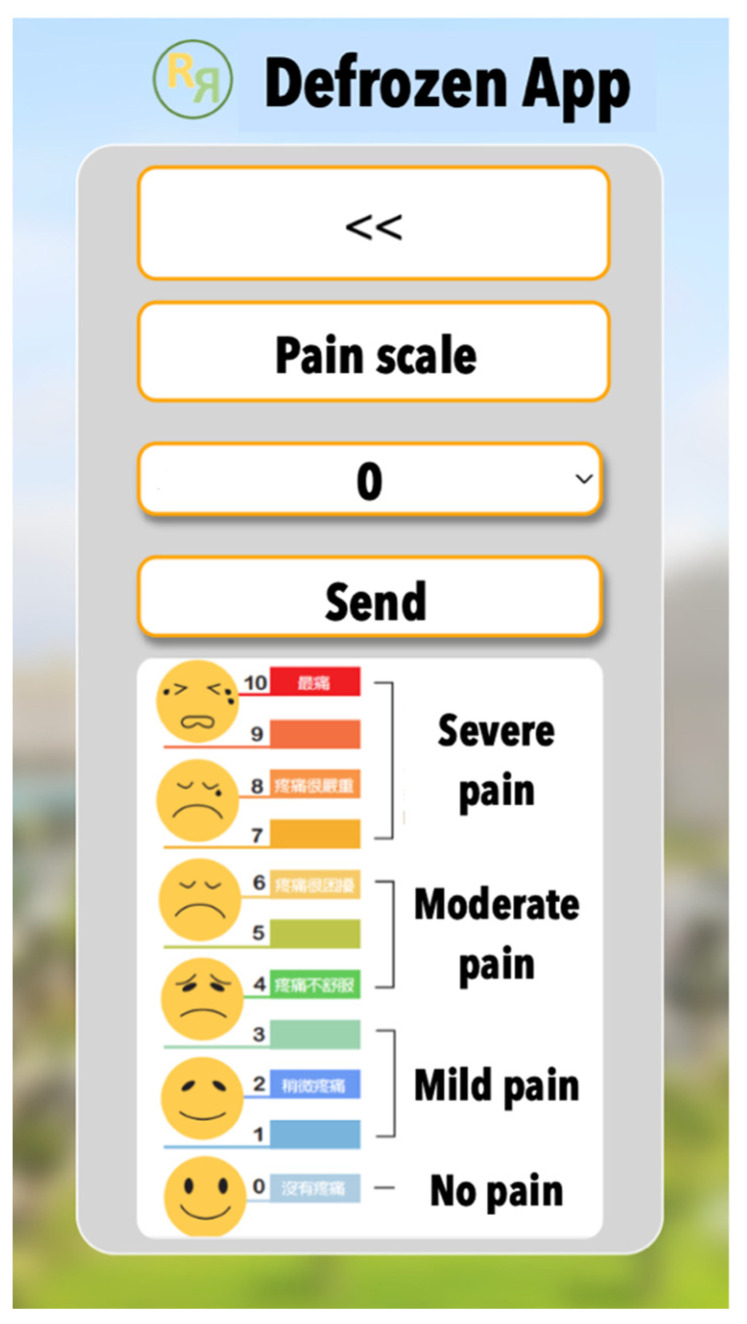
Pain scale.

**Figure 3:**
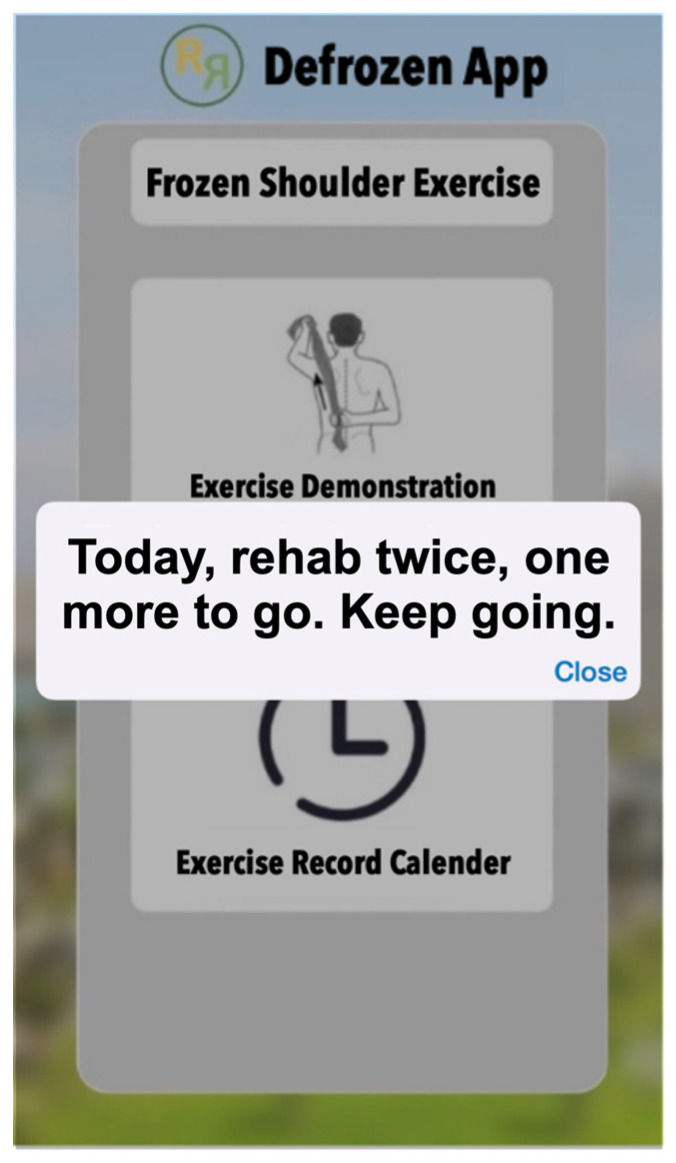
App calendar mode.

Each exercise set included 7 sets of exercise, each involving 10 repetitions, and while the app allowed for up to 4 sessions per day, we recommended participants completed at least 3 sessions daily to align with best practice guidelines [26]. Each session lasted approximately 8 min. Users were encouraged to follow the exercise demonstrations provided in the app. After completing the exercise mode, the app prompted the patient to confirm their completion and record their current pain intensity on a scale from 0 to 10 (Figure 2). Subsequently, the app transitioned to calendar mode (Figure 3), displaying the number of exercise sessions completed that day and reminding the patient of the minimum recommended sessions yet to be completed.

### 2.4. Outcome Measures

At baseline, the patient characteristics, such as age, gender, past medical history, and the side of the shoulder pain, were documented. The feasibility of the mobile application was assessed using adherence, the Technology Acceptance Model 2 (TAM2), the System Usability Scale (SUS), and the User Satisfaction and Engagement (USE) metrics after 4 weeks of interventions. 

Before and after the 4-week intervention period, relevant outcome measures, including the shoulder pain numeric rating scale (NRS), the Oxford Shoulder Score (OSS), the Quick disabilities of the arm, shoulder, and hand (QuickDASH) questionnaire, and the shoulder range of motion, were collected. 

The primary outcome measures for the feasibility included adherence, patient-reported acceptance, and the usability and safety of the Defrozen-app.

### 2.5. The Feasibility Scaling Tools

Adherence to therapy. Adherence was defined by two measures: 1. the dropout rate during the study period; and 2. the number of Defrozen-app sessions completed compared to the suggested number of exercises each day (three times every day). Exercise adherence was reported as a percentage, and adherence to the app-assisted home program was determined by the log file of the Defrozen-app. This log file enabled the computation of how many times the Defrozen-app-assisted exercise approach was followed each day.

Technology acceptance model (TAM2). To evaluate the acceptance of the Defrozen-app, participants were asked to complete a set of established questionnaires derived from the revised Technology Acceptance Model (TAM2) [19,27,28], The scoring system of TAM2 consists of three sections focusing on perceived usefulness, perceived ease of use, and intention to use, rated on a 5-point Likert scale, ranging from 1 (negative/disagree) through 3 (neutral standpoint, neither agreeing nor disagreeing), to 5 (positive/agree). 

System usability scale (SUS). The usability of the Defrozen-app was assessed using the System Usability Scale (SUS), a questionnaire designed to evaluate the usability of products and services, whether software or hardware. The SUS consists of 10 questions, each answered on a 5-point Likert scale ranging from ‘I strongly agree’ to ‘I strongly recommend disagree.’ The SUS score is presented as a percentage, ranging from 0 to 100. Typically, the average SUS score is 68, and scores up to 70 are generally regarded as a competent level of usability. A SUS score below 50 indicates a potential significant deficiency in usability, suggesting the need for substantial improvements to enhance user satisfaction. Conversely, a SUS score above 80 indicates a very usable system, suggesting high acceptability in the field [20].

User satisfaction and engagement (USE) questionnaire. The User Satisfaction and Engagement (USE) questionnaire, developed by Arnold Lund in 2001, shares a broad scope of application with the SUS. The USE questionnaire comprises items belonging to four dimensions, including usefulness, ease of use, ease of learning, and satisfaction [29]. Responses on the USE questionnaire are provided on a 5-point Likert scale, ranging from 1 (negative/disagree), through 3 (neutral standpoint, neither agreeing nor disagreeing), to 5 (positive/agree) [30].

### 2.6. Clinical Outcome Measures

Clinical outcome measures were collected both during participant recruitment and after the four weeks intervention period.

Shoulder pain intensity. Shoulder pain was assessed using the Numerical Rating Scale (NRS). Patients were instructed that a score of 0 corresponded to no pain, while a score of 10 signified the utmost level of pain. The NRS has demonstrated reliability and validity in evaluating pain among patients with shoulder conditions [31].

Oxford shoulder score (OSS). The OSS, introduced by Dawson et al. in 1996, is a patient-rated outcome measure used to assess shoulder pain and function in individuals with shoulder pain [21]. The OSS demonstrates excellent internal consistency, reliability, and validity [21,23,24], Consisting of twelve questions, the OSS provides five potential responses ranging from 0 (worst) to 4 (best) [21]. It has been employed to evaluate treatment effectiveness in cases of frozen shoulder [32].

Quick disability of the arm, shoulder, and hand (QuickDASH) score. To assess region-specific functional changes post-treatment, the QuickDASH score, developed by Beaton et al., was employed [33]. An adapted version of the original 30-item Disabilities of the Arm, Shoulder, and Hand (DASH) questionnaire, the QuickDASH consists of 11 items. Participants are required to complete a minimum of 10 items, each featuring five response options. The total QuickDASH score ranges from 0 (indicating no disability) to 100 (indicating the most severe disability) [34]. The Taiwan version of the DASH questionnaire, validated and reliable for assessing the health status of patients with upper extremity disorders, will be employed [35].

Passive range of motion (ROM) of the shoulder. The ROM of the shoulder was evaluated by a universal goniometer, measuring from specific bone landmarks, with the participants’ spine in a straight position. Passive ROM of the affected shoulder, encompassing flexion, extension, abduction, internal rotation, external rotation, and horizontal adduction will be gauged following the protocols outlined by Sharma et al. and Surena Namdari et al. [17,36], For precision, each angle was measured three times, and the mean of the three measurements was recorded. ROM of the shoulder was measured by an experienced physician with 20 years of clinical practice experience. This ensured consistent and reliable evaluations across all participants.

Statistical methods. The qualitative data were analyzed using descriptive statistics. Categorical variables will be presented as frequency and percentage, while continuous variables will be presented as mean and standard deviation. The comparison of changes before and after the intervention was conducted using Wilcoxon signed-rank test for all continuous variables. The data were entered and analyzed using STATA version 14.0 software (STATA, Inc., College Station, TX, USA).

## 3. Results

### 3.1. Participants

This prospective feasibility study initially enrolled 13 patients to evaluate the usability and acceptance of the Defrozen-app. The primary aim was to assess the feasibility of the app as a supportive tool for patients with frozen shoulder. After conducting feasibility assessments on the first three participants, the study design was adapted to include standardized clinical outcome measurements for the remaining ten patients.

The feasibility pilot study included 13 patients, consisting of six female patients and seven male patients, with a median age of 54 years old. Table 1 presents their demographic data, with six patients affected on their dominant side. 

### 3.2. Feasibility Scoring

Patients reported acceptance, usability, and satisfaction, with summarized results of the TAM-2, SUS, and USE questionnaires provided in Table 2.

#### 3.2.1. Adherence to Therapy

None of the participants dropped out from the study. The Defrozen-app program approach times per week for the thirteen patients who joined are listed in Table 3. The included patients reported a median of 2.5 sessions (range 1–5) of exercise each day during the 28 days of the intervention. Based on information obtained from the exercise log, the median adherence was 83% (range 3–100). A total of 54% (7/13) of the participants completed at least 80% of their home exercise program. For the other six patients who reported adherence to exercises of less than 80%, the main barriers were cited as time constraints (n = 3), easily forgetting (n = 2), and feeling well-recovered without needing any exercise (n = 1). Adherence patterns observed throughout the study period demonstrated a notable decline in the fourth week, with nearly half of the participants not completing any sessions.

#### 3.2.2. Technology Acceptance Model (TAM2)

The TAM2 is scored on a 0 to 5 scale. The average score for perceived usefulness was 4.5, indicating that users considered the app useful. The average score for perceived ease of use was 4.8, signifying that users found the app easy to use. The average score for intention to use was 4.4, suggesting a strong intention to further use the app.

#### 3.2.3. System Usability Scale (SUS)

The app achieved an average SUS score of 81.7 on a 0 to 100 scale. With a SUS score above 80, it indicates a very usable system, and the app may have high acceptability in the field.

#### 3.2.4. User Satisfaction and Engagement (USE) Questionnaire

The USE questionnaires are rated on a 5-point Likert scale. The users perceived the app as easy to learn, with an average score for ease of learning of 4.9. Users expressed satisfaction with the app, with an average score for satisfaction of 4.3.

### 3.3. Clinical Outcomes

The clinical outcome measurements were initially planned for ten patients. However, the data from one participant were excluded from the final analysis due to significantly limited engagement with the intervention. This participant used the app only during the first three days and completed a total of four exercise sessions. Given her minimal involvement, it was determined that including her data might not accurately reflect the intended effects of the Defrozen-app-delivered exercises following injection. This exclusion was made to ensure the integrity and reliability of the study’s findings, focusing on participants who engaged more substantially with the intervention.

For the other nine patients, the majority reported an improvement in their shoulder pain, and the pain scale showed a significant decrease (median 6 vs. 2; *p* = 0.015). The comparison of the Oxford Shoulder Score (OSS) between the initial evaluation and the 4-week intervention also showed a significant decrease (median 29 vs. 41; *p* = 0.013). The Quick DASH questionnaire was also improving after the interventions (median 34.1 vs. 13.6; *p* = 0.028), indicating an improvement in shoulder-related disability (Table 4).

In terms of the improvement in shoulder range of motion, after one month of the Defrozen-app-delivered exercise, there were significant improvements in flexion (median 130 vs. 150; *p* = 0.008), extension (median 40 vs. 58.3; *p* = 0.012), abduction (median 90 vs. 143; *p* = 0.008), internal rotation (median 45 vs. 65; *p* = 0.024), and horizontal adduction (median 100 vs. 120; *p* = 0.009) (Table 4). 

## 4. Discussion

This study provided evidence supporting the effects of home exercise after intra-articular joint corticosteroid injection in frozen shoulder patients. We developed an easy-to-use Defrozen-app for home exercise guidance with the aim of improving exercise feasibility [37]. This study revealed that the Defrozen-app is feasible and safe for patients with FS. The outcome measurements showed that the tested participants experienced reduced pain, increased shoulder range of motion, and improved shoulder functions after one month of Defrozen-app-delivered exercise. These findings may contribute to facilitating the use and implementation of digital health to improve access to shoulder exercise programs and integrate holistic interventions into plans of care for individuals with FS.

A previous study demonstrated that a mobile application improves compliance in home exercise compared to paper handouts [38]. In this study, we did not offer any phone call nor text reminder for those not performing the home exercises. Our strategy to improve adherence included a goal-setting reminder designed to remind patients how many times of exercise were left every day [39]. One participant showed inferior adherence and stated she felt well-recovered and did not need exercise. Besides this participant, others had an average adherence rate of up to 82.3%, which is considered acceptable for physical activity interventions [40]. As previously noted, adherence degraded from the first to the last week among several participants. This observation aligns with the findings of P.J.A. Nicolson et al., who identified three distinct adherence trajectories: rapidly declining adherence, gradually declining adherence, and consistently low adherence [41]. The Defrozen-app helped in identifying participants with rapidly declining or low adherence trajectories. These patterns suggest that while the app serves as a useful tool for facilitating rehabilitation exercises, additional strategies such as regular personal contact, for example by phone, may be necessary to sustain high levels of engagement and adherence [42].

None of the participants reported adverse events related to the home program. Diercks and Stevens et al. performed a study [43] comparing intensive physiotherapy and passive stretching with supervised neglect exercise, which included pendulum exercises and active exercises within the painless range and encouraged resuming all activities that were tolerated. The supervised-neglect group had a significantly better constant score at all other time points, up to and including 24 months. The authors hypothesized that intensive physiotherapy and stretching place more stress on the soft tissues, potentially stimulating the inflammatory response and fibroblast activity within the shoulder joint capsule, leading to worse outcomes. We paid close attention to this possibility, and we instructed participants using the Defrozen-app to perform self-stretching exercises to a pain-limited intensity. This might be the cause of why none of the adverse events related to this home-based exercise programs were mentioned by these patients. 

Physiotherapy was associated with improved outcomes compared with the control in the early short-term range of motion, especially external rotation [9]. A recent trial suggested that there were no significant differences in the outcomes of supervised physiotherapy combined with a home exercise program when compared with a self-directed home exercise program only in patients with primary FS after intervention [8]. A recent meta-analysis further confirmed the benefits of home exercise after intra-articular steroid injection compared to those without receiving home exercise [9]. Our results showed that the FS participants, after both intra-articular and subacromial steroid injections, demonstrated significant improvements in pain, all-direction range of motion, and shoulder functions after one month of Defrozen app-guided home exercise.

The use of mobile health applications for musculoskeletal disorders of the shoulder is rapidly growing and many are available on the Google Play Store [44]. Some studies have assessed the feasibility, usability, and effects of developed apps for the telerehabilitation of frozen shoulders [28,45,46,47,48,49]. Our study also demonstrated that our developed Defrozen-app is useful, applicable, and satisfactory for patients with frozen shoulders. We plan to modify our protocol and add phone or text reminders to improve the adherence rate to home exercise. A further randomized trial will be conducted to evaluate if the Defrozen-app-supported home exercise group shows greater improvement in clinical outcomes than the conventional home exercise group instructed through printed pamphlet instructions in patients with frozen shoulders. 

Currently, only one randomized controlled trial has evaluated the clinical effects of a developed app for FS exercises [50]. The app presented by Choi et al. included four exercise programs: shoulder flexion, cross body adduction, and sleep stretch exercises for internal rotation and external rotation. Subjects needed to hold the smartphone to do all exercises for real-time visual and auditory feedback, except for external rotation. The study’s results showed no significant effects of the developed app when compared to conventional home exercise groups in terms of pain and range of motion control in patients with frozen shoulders during a three-month follow-up. The authors suggested that the lack of impact of their developed app may be attributed to the inclusion of not all exercises in the smartphone’s real-time feedback [50]. In contrast, Defrozen-app delivers seven shoulder exercises encompassing all-direction shoulder movements, including flexion, extension, abduction, adduction, internal rotation, external rotation, and pectoral muscle stretching. Through initial instruction and demonstration of correct shoulder exercise postures from the app, we suspect that the user-friendly Defrozen-app-delivered home program can prove to be more beneficial than the traditional home program delivered by pamphlet instructions.

There are several limitations to this study that warrant mention. Firstly, the small sample size may not fully capture the broader effects of the Defrozen-app on patients with frozen shoulder (FS), although this is typical for a feasibility or pilot study [18]. Secondly, as this study was primarily designed to assess feasibility, the clinical evaluation and the improvements observed in participants cannot be attributed directly to the app’s clinical effectiveness. Therefore, the results should not be interpreted as a measure of the actual therapeutic impact of the app. Additionally, the absence of reported adverse events should be interpreted with caution due to the small sample size; a larger study might reveal safety concerns that were not apparent in this preliminary research. Our current study, with its shorter intervention duration, aligns with the objectives of a feasibility study and provides essential insights that will guide the refinement of the Defrozen-app and the design of forthcoming interventions. A future randomized controlled trial (RCT) is mandated, which should include a longer follow-up period and involve both a control group and a larger sample to rigorously test the app’s impact on patients with frozen shoulder [37]. This RCT should also carefully monitor for any adverse effects to provide a comprehensive assessment of safety as well as efficacy. The extended duration of the RCT is intended to provide a more accurate assessment of the sustained impacts of the app.

## 5. Conclusions

To conclude, our feasibility study evaluated the effectiveness of the developed mobile phone application in supporting home exercises for patients with FS. The Defrozen-app not only illustrated the correct posture for home exercises but also employed a goal-setting strategy to enhance patient compliance. Users reported high acceptance of the technology and found the app to be very user-friendly. Importantly, significant improvements were observed in key clinical outcomes, including pain intensity, shoulder range of motion, and overall shoulder function.

## Figures and Tables

**Table 1:** Patient baseline characteristics.

Demographics	Defrozen-App Participants (n = 13)
Age (years), median (IQR)	54 (IQR: 49–60)
Gender (male/female)	6:7
Dominant side involvement	6 (46%)
Duration of symptoms (months), median (IQR)	3 (IQR: 2–3)
Employment status summary	
In paid work	6 (46%)
Not in paid work	7 (54%)
Previous physiotherapy for affected shoulder	
Yes	2 (15%)
No	11 (85%)

IQR: Interquartile range.

**Table 2:** Results of the usability and acceptance questionnaires (n = 13).

Questionnaires	Score
TAM-2: Intention to use	4.4 ± 1.1
TAM-2: Perceived usefulness	4.5 ± 0.9
TAM-2: Perceived ease of use	4.8 ± 0.5
SUS	81.7 ± 15.7
USE: Ease of learning	4.9 ± 0.4
USE: Satisfaction	4.3 ± 0.6

Data represent the mean value ± standard deviation. SUS: System Usability Scale; TAM-2: Technology Acceptance Model-2; USE: Usefulness, Satisfaction, and Ease of use.

**Table 3:** Defrozen-app approach time.

ID	Approach Time (Times/Week)				Average Approach Times/Day	Adherence Rate (Suggest 3 Times per Day), %
	Week 1	Week 2	Week 3	Week 4		
1	14	14	14	14	2.0	67
2	12	17	5	0	1.2	40
3	27	24	28	30	3.9	100
4	28	28	28	28	4.0	100
5	29	25	16	0	2.5	83
6	25	16	0	0	1.5	50
7	20	21	28	25	3.4	100
8	4	0	0	0	0.1	3
9	25	26	28	28	3.8	100
10	11	7	0	0	0.6	20
11	24	25	24	23	3.4	100
12	27	26	8	0	2.2	70
13	26	20	27	27	3.6	100

ID: identification number.

**Table 4:** Secondary outcome measures: baseline vs. 4-week evaluation (n = 9).

Outcome Variables	Baseline		4-Week		*p* Value
	Median	IQR	Median	IQR	
Pain NRS	6	5–7	2	1–2	0.015
OSS	29	26–34	41	39–43	0.013
QuickDASH score	34.1	25–47.2	13.6	9–18	0.028
Range of motion of shoulder					
Flexion	130	110–132	150	135–160	0.008
Extension	40	35–50	58.3	50–62	0.012
Abduction	90	50–100	143	130–180	0.008
Internal rotation	45	10–60	65	46–67.3	0.024
External rotation	40	35–80	69	62.7–79	0.086
Horizontal adduction	100	80–102	120	116–125	0.009

IQR: interquartile range; NRS: numeric rating scale; OSS: Oxford Shoulder Score; QuickDASH score: Quick Disability of the Arm, Shoulder, and Head Score.

## Data Availability

The data presented in this study are available on request from the corresponding author.
